# Advancing Analytical Techniques in PET and rPET: Development of an ICP–MS Method for the Analysis of Trace Metals and Rare Earth Elements

**DOI:** 10.3390/foods13172716

**Published:** 2024-08-27

**Authors:** Fabiana Di Duca, Paolo Montuori, Elvira De Rosa, Bruna De Simone, Stefano Scippa, Giuseppe Dadà, Maria Triassi

**Affiliations:** 1 Department of Public Health, University “Federico II”, Via Sergio Pansini n. 5, 80131 Naples, Italy; fabianadiduca91@gmail.com (F.D.D.); derosaelvira92@gmail.com (E.D.R.); desimonebruna7@gmail.com (B.D.S.);; 2 CORIPET Consorzio Volontario, Via S. Maurilio n. 23, 20123 Milan, Italy

**Keywords:** polyethylene terephthalate (PET), recycled PET (rPET), non-intentionally added substances (NIASs), rapid analytical method, sensitive detection

## Abstract

Despite the extensive use of recycled polyethylene terephthalate (rPET) in food contact materials (FCMs), research on the presence of heavy metals (HMs) and rare earth elements (REEs) during various recycling stages (e.g., flakes, granules, and preforms) remains limited. This study aimed to address these gaps by validating a rapid and sensitive analytical method to quantify 26 HMs and 4 REEs in PET and rPET matrices. An ICP-MS method was validated per EURACHEM guidelines, assessing linearity, limits of detection (LOD), limits of quantification (LOQ), accuracy, and repeatability. The method was employed for initial screening of HMs and REEs classified as non-intentionally added substances (NIASs) in PET and rPET samples. The findings showed high accuracy and reliability, with recovery rates between 80% and 120%. Analysis revealed varying concentrations of HMs and REEs, with the highest levels in 100% rPET preforms, notably Zn, Cu, and Al among HMs, and La among REEs. The study identified critical contamination points during the recycling process, highlighting the need for targeted interventions. This research provides a crucial analytical framework for assessing HMs and REEs in PET and rPET, ensuring FCM safety compliance and supporting efforts to enhance rPET product safety, promoting public health protection and advancing the circular economy.

## 1. Introduction

The current global production trends, coupled with a growing demand for convenience foods, have led to the widespread processing, storage, and packaging of food using food contact materials (FCMs) [1]. According to the European legislation, FCMs include all materials and their respective articles intended to come into direct or indirect contact with food, such as transport containers, production machinery, packaging, or kitchen equipment [2,3,4]. Currently, plastics, which are typically manufactured from oil or petroleum composed of monomers and chemically modified basic components, represent one of the largest end-use markets [5]. Particularly, in Europe, the polyethylene terephthalate (PET), comprising 7.9% of the market share in 2019, stands out as a prevalent choice for beverage packaging [6]. 

PET is a recyclable choice, providing performance benefits unmatched by alternative options [7]. It replaces glass primarily, but also metal cans [8], owing to its outstanding attributes such as higher quality of transparency, lightweight composition, effective barriers against gas and water, impact resistance, UV stability, and durability [9,10]. The use of recycled PET (rPET) for FCMs represents progress toward a circular economy, as PET’s recyclability facilitates its reuse for various purposes [11]. Waste can be transformed into reusable containers, re-enter the waste stream, recover energy, and be recycled for durable applications [12,13]. However, there is a lack of a comprehensive analytical framework to assess end-of-life and recycling processes. Safety evaluation currently relies on limited testing, but there is a need for broader measures covering migrating substances, non-intentional additives, contaminants, and polymeric degrading compounds to ensure compliance with safety standards for rPET usage [14]. 

Several studies have focused on the post-consumer phase of rPET products, particularly regarding the potential contamination risk associated with packaging polymers [13]. Additionally, growing public consciousness regarding contamination in FCMs has sparked worldwide apprehension over potential environmental pollution upon their disposal post-use [15]. Indeed, several thousand chemicals known intentionally and unknown non-intentionally added substances are used in the manufacturing of FCMs [16]. Particularly, the non-intentionally added substances (NIASs) are usually formed as by-products or degradation products during the production or recycling, or can be attributed to impurities in the starting materials used [17]. 

Heavy metals (HMs), classified as trace elements and environmental contaminants, may be present in PET/rPET materials and persist as NIASs in the final food contact articles (FCAs), potentially migrating into food or beverages and thereby posing serious health concerns [18,19,20,21]. The presence of HMs in recycled products, including both intentionally added substances (IASs) and non-intentionally added substances (NIASs), is a major issue for rPET used in food contact applications [20]. Certain metals are deliberately incorporated during manufacturing as additives, like colorants, antioxidants, and stabilizers [22,23,24,25]. Additionally, HMs such as Pb, Cd, Cr, Hg, Ni, can enter the recycling stream from packaging materials, recycling equipment, or the original materials [15,17,26]. Detecting trace metals in PET/rPET materials like flakes, granules, and preforms is crucial because of the potential health hazards. In addition to HMs, rare earth elements (REEs) can be found in rPET due to contamination during the recycling process, often originating from electronic waste or other materials containing rare earths inadvertently mixed with plastic waste [27]. This raises concerns about their potential release into the environment or contamination of the recycling chain if not properly addressed [28,29,30]. Furthermore, the EFSA Panel on Food Contact Materials, Enzymes and Processing Aids (CEP Panel) assessed the safety of the additive Ln 1,4-benzene dicarboxylic acid (with Ln = La, Eu, Gd, Tb), stating that it does not raise a safety concern for the consumer under the proposed conditions of use and if the migration of the sum of the four lanthanides in ionic form does not exceed 50 μg/kg food [31]. However, the EU Regulation 2020/1245 has introduced substantial amendments to EU Regulation 10/2011 concerning specific standards for materials and articles made of plastic intended to come into contact with food products (FCMs) [2,4]. The ongoing pursuit of ever-increasing food safety levels prompts the European Authority to periodically review contaminant levels and/or migration limits. Indeed, EU Regulation 2020/1245 was published precisely to revise certain limits, establishing a higher level of safety, and it has authorized the use of compacts in the manufacture of plastic materials and articles intended for food contact [2,4].

Analytical techniques are essential for identifying HMs and REEs in rPET materials such as flakes, granules, and preforms, ensuring the safety and regulatory compliance of PET packaging in the food industry. To achieve this, analytical methods must be validated to verify their specificity, accuracy, and reproducibility within the relevant concentration range [32]. Presently, various analytical techniques are employed to assess consumer and environmental exposure to heavy metals originating from the utilization of polymeric substances in FCMs [15]. However up to date these analytical techniques are, there is no consensus on sample preparation methods used to digest and analyze rPET packaging for HMs and REEs content to comply with the European legislation [15]. Therefore, the identification and analysis of NIASs, including HMs and REEs in FCMs, is already a big challenge since detecting hazardous compounds in such materials can be a complex task [21,33]. However, as widely reported in the literature, the identification and quantification of HMs and REEs in PET/rPET matrices is performed through acid digestion, typically conducted using strong acids such as sulfuric acid (H_2_SO_4_), nitric acid (HNO_3_), and/or hydrochloric acid (HCl). These acids are used either individually or in combination [15]. For example, Nguyen et al. validated a method for accurately detecting and measuring trace levels of heavy metals, including cobalt, germanium, arsenic, and others, in packaging materials commonly used in the food industry, such as PET. Particularly, they performed the acid digestion by adding first nitric acid (65%) and then hydrochloric acid [34]. In an earlier study, the research by Takahashi et al. documented a high-temperature acid digestion protocol using sulfuric acid for the quantification of Sb in PET [35]. In the same year, Westerhoff et al. outlined a method for digesting PET from water bottles using an HNO_3_/HCl acid solution at 180 °C for 15 min under 250 psi pressure [36]. Currently, there are no studies in the literature that describe a comprehensive evaluation of all HMs in PET materials, and furthermore, studies that have evaluated NIASs in rPET FCMs are still limited. Most studies have focused on the presence or migration of organic contaminants, which can degrade or migrate during use and recycling. In contrast, inorganic substances such as metals generally persist in the material even after recycling [23]. However, antimony (Sb) has been analyzed extensively in PET/rPET as it is a common polymerization catalyst [35,36,37,38,39]. Although other metals, such as Pb, Cd, Cr, As, and Mn have been investigated less frequently [15,17].

In light of the aforementioned, the study’s objective was to close any gaps in the assessment of trace elements in PET and rPET by validating a highly sensitive and quick analytical method for the simultaneous quantitative determination of 26 HMs and 4 REEs in PET and rPET matrices. Specifically, the study involved: (i) the evaluation of linearity, limits of detection (LOD), limits of quantification (LOQ), accuracy, and repeatability through recovery tests in both PET and rPET matrices, and (ii) the application of the validated method on real PET and rPET samples for the identification and quantification of NIASs (HMs and REEs). 

## 2. Materials and Methods

### 2.1. Reagents and Standard Solutions 

Nitric acid (HNO_3_, 65%) and hydrochloric acid (HCl 37%) of ultra-trace analysis grade were purchased from Merck (Merck, Darmstadt, Germany). Deionized water (18.2 MΩ) was procured using a Milli-Q purification system (Millipore Direct-Q UV, Bedford, MA, USA). Multi-element reference solutions for inductively coupled plasma mass spectrometry (ICP-MS) were used for preparation of the calibration standards. In detail, the multielement standard solution (1000 µg/mL) containing Al, Sb, As, Ba, B, Cd, Ca, Co, Cr, Fe, Ge, Li, Mg, Mo, Ni, Pb, Cu, Se, Sn, Sr, V, and Zn, the multielement standard solution (1000 µg/mL) containing Ca, K, and Mg, and the standard solution of Hg (10 µg/mL) were purchased from Sigma-Aldrich (St. Louis, MO, USA). The internal standard solution for ICP-MS was prepared by mixing and diluting standard solutions of In, Ir, and Y (Sigma Aldrich, St. Louis, MO, USA) containing elemental concentrations of 100 µg/mL. The standard solution (1000 µg/mL) of La, Eu, Gd, and Tb were purchased from Merck (Merck, Darmstadt, Germany). 

### 2.2. Sample Preparation

To conduct a screening for potential HMs and REEs in PET/rPET, 0.1 g of samples (preforms were cut into pieces with dimensions of 1 × 1 cm) were weighed into a quartz vessel and underwent acid digestion. Specifically, an acid digestion method has been optimized for the complete dissolution of both PET and rPET samples using a combination of strong acids, which ensures the breakdown of the polymer matrix and the release of target metal ions. The acid digestion was carried out by 2 mL of 98% sulfuric acid overnight, followed by 5 mL of HNO_3_ and HCl mixture (3:1, *v*/*v*). The mixture stood at room temperature for 30 min before being heated to 280 °C for 1 h to ensure complete digestion. Subsequently, the digested samples were cooled to room temperature, transferred to metal-free calibrated flasks for ICP/MS analysis, and adjusted to a volume of 20 mL with deionized water. The samples were subjected to quali-quantitative analysis using ICP-MS, which allows for highly sensitive and accurate detection of trace elements. This approach not only facilitates the monitoring of contaminant levels in this matrix but also ensures compliance with environmental and safety regulations, thereby supporting sustainable recycling practices.

### 2.3. HMs and REEs Analysis by ICP-MS

The quali-quantitative analyses were performed by Thermo Scientific^TM^ ICAP^TM^ RQ inductively coupled plasma mass spectrometry (ICP-MS) (Waltham, MA, USA), which employed the Thermo Scientific^TM^ Qtegra^TM^ Intelligence Scientific Data Solution^TM^ (ISDS) Software (V. 2.10.3324.131) (the details regarding the studied analytes are provided in Table S1). The resolution adjustment and mass calibration were performed on a daily basis utilizing the AutoTune function to ensure the stability of the instrument sensitivity. Therefore, the instrument was tuned prior to analyses using the kinetic energy discrimination (KED) mode in the collision cell (QCell^TM^), utilizing Argon as the collision gas. Additionally, the instrument featured a Teledyne CETAC Technologies ASX-560 autosampler. A tune solution containing Ba, Bi, Ce, Co, In, Li, and U at a concentration of 1.00 ± 0.05 µg/L in a 2% (*v*/*v*) HNO_3_ and 0.5% (*v*/*v*) HCl aqueous solution was used. The criteria required for successful tuning are summarized in Table S2. This solution was supplied by the manufacturing company, and it was utilized for instrument calibration prior to conducting an analysis. Each sample was evaluated in triplicate, with data provided as mean concentration ± standard deviation (SD). 

### 2.4. Standard Solutions 

The internal standard mixture was prepared with each component at a concentration of 100 µg/L by dilution of stock solutions with HNO_3_ (1% *v*/*v*) in deionized water. Internal standards were introduced seamlessly into the instrument throughout the analytical process, utilizing an automated online internal standard addition system. Particularly, this solution was mixed with blanks, calibration solutions, and samples using a peristaltic pump at a fixed rate (4:1). For target analytes, standard solutions were prepared through the appropriate dilutions of their stock solutions using HNO_3_ (1% *v*/*v*) as the diluent. In detail, standard solutions used for calibration curves were prepared with concentrations in the range of 0.1–100.0 µg/L for As, Ba, Cd, Co, Cr, Ge, Mo, Sr, and V and 0.2–200.0 µg/L for B, Li, Mn, Hg, Ni, Pb, Cu, Se, Sn, and Zn. In addition, the calibration curves were in the range of 0.5–200.0 µg/L for Al and Fe, 0.1–500.0 µg/L for Sb, and 1.0–500.0 µg/L for Ca, Mg, K, and Na. Furthermore, for rare earth elements (La, Eu, Gd, and Tb) the calibration curves ranged from 0.2 µg/L to 200.0 µg/L. 

### 2.5. Quality Assurance and Quality Control

The method validation followed the protocol from EURACHEM guidelines [34,40,41]. Therefore, according to the above guidelines, the performance characteristics evaluated during method validation were the working range, including linearity, the LOD, LOQ, and the accuracy and repeatability. 

Acceptance criteria for linearity were considered met when the correlation coefficient (R^2^) was higher than 0.990. Instead, for recovery tests, the criteria were set at 80–120% for accuracy and repeatability. The normality of the data distribution was assessed using the Shapiro–Wilk test at a significance level of 95%, which is suitable for sample sizes ranging from 3 to 50. To identify and eliminate potential outliers, the Dixon and Grubbs criteria were applied. The Dixon criterion, valid for sample sizes up to 40, helps to detect the presence of a single extreme value, either too high or too low. Meanwhile, the Grubbs criterion is capable of determining if the two highest or two lowest values in a dataset can simultaneously be considered outliers.

### 2.6. Real Samples

**Figure 1:**
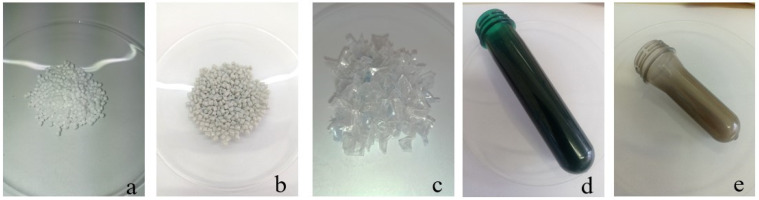
(**a**) Virgin PET granules; (**b**) recycled PET granules; (**c**) recycled PET flakes (**d**) preform 50% vPET/50% rPET; (**e**) preform 100% rPET.

Five varied sample types, comprising granules, pellets, and preforms made of both PET and rPET, were randomly collected from a company within the EU. In accordance with a confidentiality agreement, the names of the manufacturers are not disclosed. Samples were preserved in metal-free falcon tubes or in acid-washed plastic bags until analysis. The sample types analyzed are visually depicted in Figure 1. 

## 3. Results and Discussion

### 3.1. Optimization of ICP-MS

The instrumental optimization was carried out through adjustments to the nebulizer gas and make-up gas flows to ensure plasma stability. This was achieved by fine-tuning the torch position and minimizing the formation of oxides and doubly charged ions using the standard calibration solution. The optimized instrumental parameters employed during the analysis are listed in Table 1.

### 3.2. Method Quality Assurance

#### 3.2.1. Linearity, LOD, and LOQ

The linearity was determined by evaluating the correlation between different analyte concentrations and their corresponding instrumental responses. In detail, calibration standards for 26 HMs (Al, Sb, As, Ba, B, Cd, Ca, Co, Cr, Fe, Ge, Li, Mg, Mn, Hg, Mo, Ni, Pb, K, Cu, Se, Na, Sn, Sr, V, and Zn) and 4 REEs (La, Eu, Ga, and Tb) were prepared and analyzed using ICP-MS. For instance, for Al and Fe, concentrations of 0.5, 1.0, 2.5, 5.0, 25.0, 50.0, 100.0, and 200.0 µg/L and of 0.1, 0.5, 1.0, 2.5, 5.0, 25.0, 50.0, 100.0, 250.0, and 500.0 for Sb were utilized. Furthermore, for As, Ba, Cd, Co, Cr, Ge, Mo, Sr, and V, linear ranges were evaluated through 8 calibration points (0.1, 0.5, 1.0, 2.5, 5.0, 25.0, 50.0, and 100.0 µg/L), while 9 calibration points were used for B, Li, Mn, Hg, Ni, Pb, Cu, Se, Sn, and Zn (0.2, 0.5, 1.0, 2.5, 5.0, 25.0, 50.0, 100.0, and 200.0 µg/L). For the remaining analytes, the curves were constructed using 8 calibration points (0.1, 0.5, 1.0, 2.5, 5.0, 25.0, 50.0, and 100.0 µg/L for La, Eu, Gd, and Tb and 1.0, 2.5, 5.0, 25.0, 50.0, 100.0, 250.0, and 500.0 µg/L for Ca, Mg, K, and Na). As shown in Table 2, for all analytes, the correlation coefficients (R^2^) met the acceptability criteria (>0.990). These results indicated the linearity of the method within the measurement ranges. Additionally, Table 2 also provides the LOD and the LOQ obtained for the studied analytes. The LOD and the LOQ were in the range of 0.006–0.030 mg/kg and 0.02–0.20 mg/kg, respectively. Some spectra obtained from the instrumental analysis and the graphical recovery of IS are provided in the Supplementary Material (Figures S1–S5). 

#### 3.2.2. Accuracy and Repeatability

Repeatability refers to the consistency of independent test results obtained using the same method, material, operator, and equipment within a short time frame. Accuracy, on the other hand, indicates how closely the measured values align with the true or accepted reference values, reflecting how precisely the method measures the actual concentration or quantity of the analyte in the sample [34,42]. 

To evaluate the repeatability and the accuracy of the analytical method and to ensure that no losses or contamination occurred during the entire analytical process, recovery tests on fortified samples were conducted. Specifically, virgin PET (Figure 1a) and recycled PET granules (Figure 1b) were spiked at three different levels of analyte concentrations and then subjected to the entire digestion and instrumental analysis process. The mean values, standard deviations (SDs), relative standard deviations (%RSDs), and recoveries (%Rec) were calculated. The recoveries were computed according to Equation (1) [34]:

(1) $$\%Rec=\frac{\left(C_{spiked}-C_{ref}\right)}{C_{add}}\times 100$$

where C_spiked_ (mg/kg) is the spiked concentration of each analyte, C_ref_ (mg/kg) is the concentration of the analyte in the sample unspiked, and C_add_ (mg/kg) represents the concentration of the analyte used to fortify the sample.

In detail, the repeatability and accuracy were evaluated at 0.10, 5.00, and 40.00 mg/kg for Al and Fe, at 0.02, 10.00, and 100.00 mg/kg for Sb, at 0.02, 1.00, and 20.00 for As, Ba, Cd, Co, Cr, Ge, Mo, Sr, V, La, Eu, Ga, and Tb, at 0.04, 5.00, and 40.00 for B, Li, Mn, Hg, Ni, Pb, Cu, Se, Sn, and Zn, at 0.20, 10.00, and 100.00 for Ca, Mg, K, and Na. The findings of the method validation for repeatability are presented in Table 3 and Table 4, with calculated analyte concentrations (as mean values), standard deviations (SDs) and relative standard deviations (RSDs (%)). 

For all the analytes studied, the RSD% results were below 20%, thereby meeting the proposed acceptability criteria. Specifically, for virgin PET samples (Table 3), the RSD% ranged from 1.3% to 19.8%, and for the rPET samples (Table 4), from 4.3% to 15.7%. These findings confirm that the developed methodology for determining HMs and REEs in PET and rPET matrices is repeatable.

**Figure 2:**
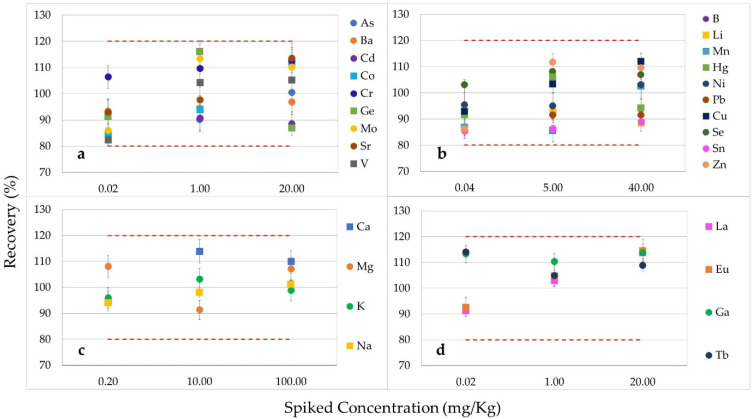
Percentage recoveries (on the y-axis) obtained for (**a**) As, Ba, Cd, Co, Cr, Ge, Mo, Sr, and V; (**b**) B, Li, Mn, Hg, Ni, Pb, Cu, Se, Sn, and Zn; (**c**) Ca, Mg, K, and Na; (**d**) La, Eu, Ga, and Tb at three different spiked levels (on the x-axis), with the relative standard deviations (RSDs) as error bars in virgin PET samples.

**Figure 3:**
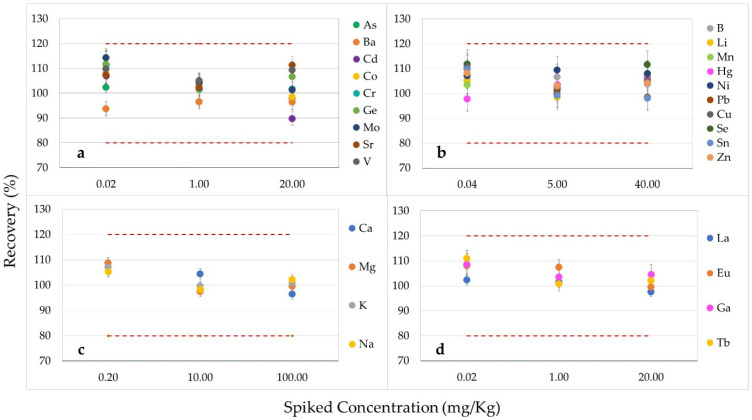
Percentage recoveries (on the y-axis) obtained for (**a**) As, Ba, Cd, Co, Cr, Ge, Mo, Sr, and V; (**b**) B, Li, Mn, Hg, Ni, Pb, Cu, Se, Sn, and Zn; (**c**) Ca, Mg, K, and Na; (**d**) La, Eu, Ga, and Tb at three different spiked levels (on the x-axis), with the relative standard deviations as error bars (RSDs) in rPET samples.

Figure 2 and Figure 3 present the accuracy results from the method validation, expressed as percentage recoveries, for (a) As, Ba, Cd, Co, Cr, Ge, Mo, Sr, and V; (b) B, Li, Mn, Hg, Ni, Pb, Cu, Se, Sn, and Zn; (c) Ca, Mg, K, and Na; (d) La, Eu, Ga, and Tb at three different spiked levels with the relative standard deviations as error bars (RSDs) in vPET samples and in rPET samples, respectively. In detail, the mean recoveries for all studied analytes were in the range of 82.3% (recorded for V at the lowest level of validation)—116.1% (detected for Ge at the intermediate level of validation) for virgin PET samples, and ranged from 89.9% (found for Cd at the highest level of validation)—114.3% (detected for Mo at the lowest level of validation) for rPET samples. The high recovery rates indicate that the method achieved accuracy without interferences. Consequently, the results met the study’s acceptability criteria, confirming the method’s accuracy for determining analytes both in the PET and rPET matrices.

Based on the method validation results, it can be concluded that the linearity and recovery assessments met all established acceptance criteria, confirming the analytical method’s robustness and reliability. The linearity analysis showed a correlation coefficient (R^2^) consistently above 0.990, indicating a strong linear relationship and reliable measurements across the tested range. Recovery tests demonstrated accuracy and repeatability within the acceptable range of 80–120%, verifying the method’s precision and accuracy. This consistency in yielding results close to the true value underscores the method’s reliability.

### 3.3. Application of the Method to Real Samples

**Figure 4:**
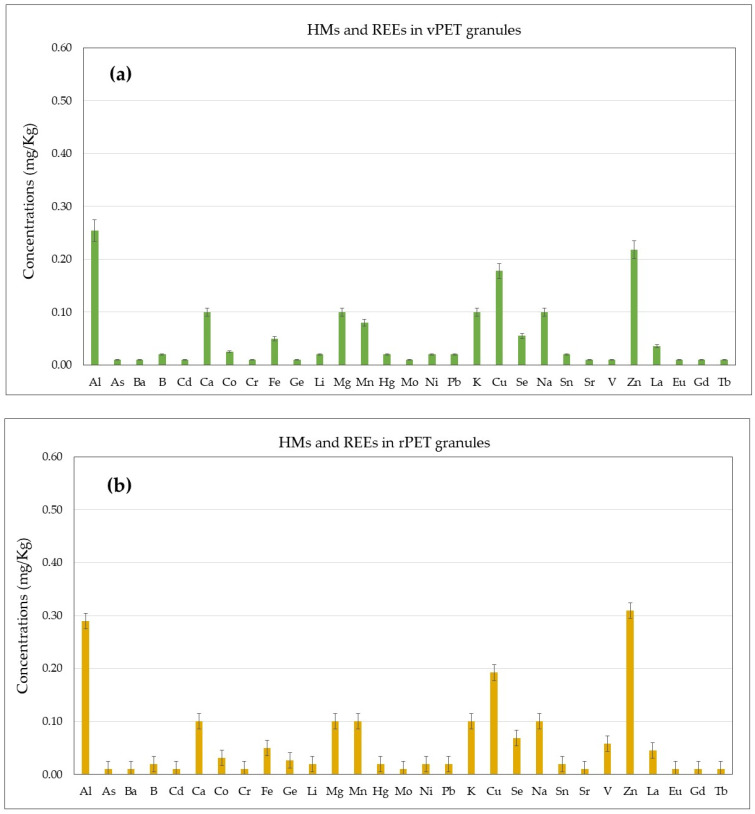

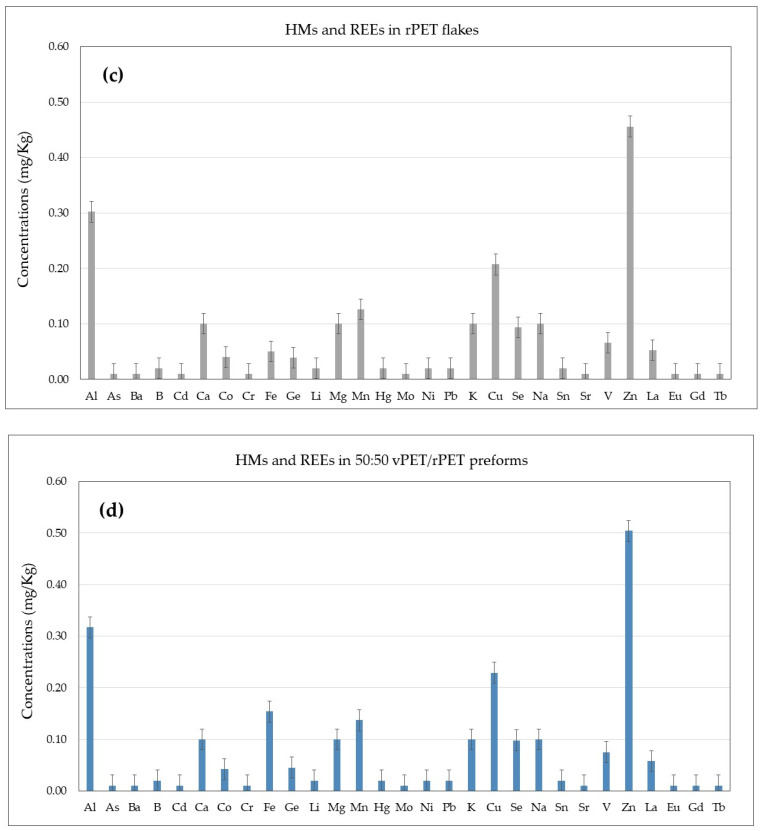

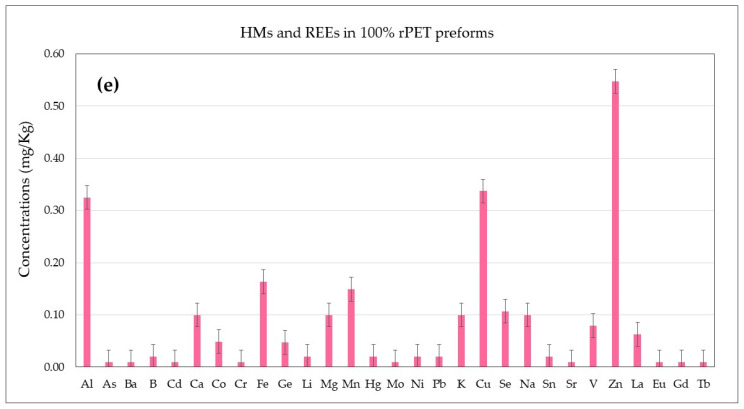
Concentrations (mg/kg) of 26 HMs and 4 REEs assessed in (**a**) vPET granules; (**b**) rPET granules; (**c**) rPET flakes; (**d**) 50:50 vPET/rPET preforms; and (**e**) 100% rPET preforms.

The developed method has been utilized to quantify 26 HMs and 4 REEs in PET and rPET samples (Figure 1). The results are shown in Figure 4. In detail, Figure 4 illustrates the concentrations of HMs and REEs (mg/kg) found in vPET granules (Figure 4a), rPET granules (Figure 4b), rPET flakes (Figure 4c), 50:50 vPET/rPET preforms (Figure 4d), and 100% rPET preforms (Figure 4e).

In detail, total amounts ranged from 1.53 mg/kg to 2.27 mg/kg, with a minimum total amount recorded for the vPET granules matrix (Figure 4a) and the highest amount detected for 100% rPET preforms (Figure 4e). Particularly, as showed in Figure 4e, the highest amount was found for Zn (0.547 mg/kg), followed by Cu (0.337 mg/kg) and Al (0.325 mg/kg). Specifically, the mean concentrations ranged between 0.254 mg/kg and 0.325 mg/kg for Al, 0.025 mg/kg and 0.049 mg/kg for Co, 0.080 mg/kg and 0.149 mg/kg for Mn, 0.178 mg/kg and 0.337 mg/kg for Cu, 0.055 mg/kg and 0.107 mg/kg for Se, 0.218 mg/kg and 0.547 mg/kg for Zn. The presence of metals such as Al, Zn, and Cu in FCMs made from recycled PET can be traced back to several factors, including the recycling process itself and the material’s origin. Accordingly, during recycling, PET may come into contact with trace metals through equipment or surfaces that contain them, such as recycling lines or machinery with metal components [29,43]. Therefore, several previous studies have reported that Zn and Al offer superior catalyst performance compared to other transition metal catalysts [9,44]. Instead, the presence of Cu could be due to the use of colorants, since copper phthalocyanine blue is often applied as a colorant for food packaging and containers [45]. For the group of REEs, the only quantifiable concentrations were those of La, with the highest levels (0.063 mg/kg) found in the 100% rPET preform matrix (Figure 4e). For the other REEs, all the results were below the limit of quantification. However, interestingly, for the analytes with quantifiable concentrations of HMs, the levels showed an increasing trend in the following order: vPET granules > rPET granules > rPET flakes > 50:50 vPET/rPET preforms > 100% rPET preforms. Recycled PET could be more contaminated than virgin PET due to several factors [46,47]. Throughout its lifecycle, PET can absorb metal traces from non-food industrial applications and container coatings. Additionally, the recycling process may introduce contaminants through the use of contaminated water or detergents [18]. Indeed, some additives or colorants used in PET production may contain traces metals, which can be released during the recycling process. Repeated exposure to heat and light during the PET lifecycle and recycling process can degrade the material, leading to the release of metals that were previously bound within the polymer matrix [20]. Furthermore, if the recycled PET comes from non-food industrial applications, it might have absorbed metal traces from industrial processes or containers with metal coatings. Finally, since the Sb concentrations were significantly higher than those of the other analytes, it was excluded from Figure 4 to allow for a clearer and more immediate assessment of the levels of the other analytes, which are less studied in the literature. However, the concentrations of Sb ranged from 56.25 mg/kg (found in virgin PET granules—Figure 1a) to 77.83 mg/kg (found in 100% rPET preforms—Figure 1e). The presence of Sb in PET materials can be explained by its role as a catalyst in PET production, where antimony (Sb_2_O_3_) is used for this purpose, as stated by Duh et al., who documented an Sb content in commercial PET resins in the range of 190 mg/kg to 300 mg/kg [48]. Similarly, other studies have reported Sb concentrations in PET catalyzed with Sb that are higher than those observed in this study, ranging from 150 mg/kg to 300 mg/kg [15,25,37,38].

As previously mentioned, there are currently few studies in the literature that have assessed the levels of the analytes examined in this research, as most of them have focused on the study of contaminant migration from FCMs [17,19,21,26,34,38,49,50]. However, only few studies have delved into the levels of HMs in both vPET and rPET, and the few available scientific studies pertain to bottles. In a recent study, Chibwe et al. employed a comprehensive screening approach to evaluate inorganic chemicals in plastic flakes and recycled pellets used in the production of recycled goods, including PET products [30]. Specifically, the research conducted by Chibwe et al. revealed that the total concentration of 60 HMs ranged from 0.005 mg/kg to 2980 mg/kg, with the highest levels found for Ca (2980 mg/kg), Na (617 mg/kg), and Fe (156 mg/kg). In the same study, 7 REEs, including La, Ga, and Eu, were detected in at least one sample with amounts in the range of <LOQ–0.406 mg/kg [30]. The presence of REEs in plastics is thought to stem from recycled materials originating from electrical and electronic equipment. As a result, these substances have been suggested as indicators for monitoring unintended contamination from electronic waste [29]. Additionally, Curtzwiler et al. reported Pb and Cd levels below the LOD (0.005 mg/kg; both Pb and Cd) in virgin and recycled PET blends, while Cr was determined to be between 5 and 31 mg/kg, depending on the recycled PET concentration [51]. The results of this study suggest that recycled PET products may have higher concentrations of certain HMs compared to their virgin counterparts, as indicated by previous studies [23,30]. Specifically, this study observed that the concentrations in recycled PET were nearly double those found in virgin PET. This discrepancy is attributed to the accumulation of contaminants during the recycling process, underscoring potential challenges in maintaining the purity of recycled PET materials [23,51]. Furthermore, the research by Whitt et al. quantified Ni, Cr, Cd, Sb, and Pb in recycled PET, estimating that out of 200 samples, 29 were contaminated with HMs, with Cr and Cd present in all contaminated samples. Whitt et al. observed that PET food containers produced from post-consumer materials were contaminated with HMs, likely due to the recycling and sorting processes, which may include commingling with electronic waste [48].

The results of this study emphasize the necessity for rapid and sensitive methodologies to identify and quantify even trace contaminants in FCMs. This capability is essential for identifying critical points where contamination may occur during the recycling process. Implementing such methodologies will improve the detection of potential contaminants, leading to better quality control and safer recycled PET products. The importance of rigorous quality control measures in the recycling industry is necessary to mitigate the risk of HMs leaching from packaging materials, emphasizing the need for sustainable and safe practices in the production and use of recycled PET. These insights contribute to ongoing efforts aimed to enhance the environmental impact and safety of packaging materials within the framework of the circular economy. 

## 4. Conclusions

The development of a highly sensitive and rapid inductively coupled plasma mass spectrometry (ICP-MS) method for the simultaneous quantitative determination of 26 heavy metals (HMs) and 4 rare earth elements (REEs) in PET and rPET matrices represents a significant advancement in analytical techniques. To date, there have been very few studies examining the presence of HMs and REEs as NIASs in PET and rPET matrices, particularly across different stages of recycled material production such as flakes, granules, and preforms. This research gap is critical, as it hinders comprehensive safety evaluations of rPET used in FCMs. Assessing HMs and REEs in these matrices is crucial for public safety, as it enables the identification of key contamination points during the recycling process. By detecting and quantifying these contaminants at various stages, from initial recycling steps to the final product, this study offers valuable insights into the specific points where contamination may arise. Such information is crucial for devising targeted interventions to reduce contamination risks, ensuring that rPET materials comply with stringent safety standards and do not pose health risks to consumers. This research highlights the need for detailed analytical frameworks to safeguard public health by preventing the migration of hazardous substances from rPET packaging into food and beverages. 

## Figures and Tables

**Table 1:** Instrumental parameters of ICP-MS during analysis of HMs and REEs.

Parameter	Value
Pump Speed	30 rpm
RF Power	1550 W
Nebulizer Gas Flow rate	1.17 L·min^−1^
Plasma Gas Flow	4.8 mL min^−1^
QCell Bias	−18 V
Quadrupole Bias	−21 V
Scan Settings	0.01–0.3 s dwell time per analyte, 10 sweeps

**Table 2:** List of studied analytes with internal standard (IS) used for each of them for the ICP-MS analysis carried out in KED mode, the range of instrumental calibration curves in µg/L with correlation coefficients (R^2^), the LOD, and the LOQ.

Analyte		IS	Linearity Range (µg/L)	R^2^	LOD (mg/kg)	LOQ (mg/kg)
HMs	Al	^89^Y	0.50–200	0.992	0.030	0.10
	Sb	^115^In	0.10–500	0.997	0.006	0.02
	As	^89^Y	0.10–100	0.995	0.006	0.02
	Ba	^115^In	0.10–100	0.995	0.006	0.02
	B	^89^Y	0.20–200	0.991	0.012	0.04
	Cd	^115^In	0.10–50	0.998	0.006	0.02
	Ca	^89^Y	1.00–500	0.996	0.060	0.20
	Co	^89^Y	0.10–100	0.998	0.006	0.02
	Cr	^89^Y	0.10–100	0.998	0.006	0.02
	Fe	^89^Y	0.50–200	0.993	0.030	0.10
	Ge	^89^Y	0.10–100	0.995	0.006	0.02
	Li	^89^Y	0.20–200	0.994	0.012	0.04
	Mg	^89^Y	1.00–500	0.996	0.060	0.20
	Mn	^89^Y	0.20–200	0.992	0.012	0.04
	Hg	^115^In	0.20–200	0.995	0.012	0.04
	Mo	^89^Y	0.10–100	0.994	0.006	0.02
	Ni	^89^Y	0.20–200	0.992	0.012	0.04
	Pb	^115^In	0.20–200	0.998	0.012	0.04
	K	^89^Y	1.00–500	0.993	0.060	0.20
	Cu	^89^Y	0.20–200	0.991	0.012	0.04
	Se	^89^Y	0.20–200	0.999	0.012	0.04
	Na	^89^Y	1.00–500	0.994	0.060	0.20
	Sn	^115^In	0.20–200	0.993	0.012	0.04
	Sr	^115^In	0.10–100	0.995	0.006	0.02
	V	^89^Y	0.10–100	0.997	0.006	0.02
	Zn	^193^Ir	0.20–200	0.992	0.012	0.04
REEs	La	^115^In	0.10–100	0.995	0.006	0.02
	Eu	^115^In	0.10–100	0.997	0.006	0.02
	Gd	^115^In	0.10–100	0.998	0.006	0.02
	Tb	^115^In	0.10–100	0.994	0.006	0.02

IS: Internal Standard.

**Table 3:** Method validation results for repeatability with calculated analyte concentration (as mean values, mg/kg), standard deviations (SD) and relative standard deviations (RSD%) obtained for virgin PET samples at three spiked levels.

Analyte	Spiked Value (mg/kg)	Calculated Analyte Concentration (mg/kg)	SD (mg/kg)	RSD (%)
Al	0.10	0.105	0.017	15.9
	5.00	4.43	0.23	5.3
	40.00	38.09	3.48	9.1
Fe	0.10	0.097	0.019	19.6
	5.00	4.68	0.61	13.1
	40.00	38.23	3.03	7.9
Sb	0.02	0.018	0.003	15.6
	10.00	8.69	0.17	2.0
	100.00	101.66	3.90	3.8
As	0.02	0.019	0.003	14.9
	1.00	0.90	0.07	7.2
	20.00	20.09	3.01	15.0
Ba	0.02	0.019	0.004	19.2
	1.00	0.94	0.10	10.7
	20.00	19.35	3.17	16.4
Cd	0.02	0.017	0.001	3.0
	1.00	0.91	0.12	13.3
	20.00	17.71	0.62	3.5
Co	0.02	0.017	0.001	4.9
	1.00	0.94	0.11	11.7
	20.00	22.43	1.12	5.0
Cr	0.02	0.021	0.003	12.4
	1.00	1.10	0.12	11.0
	20.00	22.48	1.48	6.6
Ge	0.02	0.018	0.003	14.9
	1.00	1.16	0.05	4.7
	20.00	17.36	0.89	5.1
Mo	0.02	0.017	0.002	11.1
	1.00	1.13	0.21	18.9
	20.00	22.03	2.11	9.6
Sr	0.02	0.019	0.003	13.7
	1.00	0.98	0.17	17.1
	20.00	22.71	1.45	6.4
V	0.02	0.016	0.001	7.9
	1.00	1.04	0.12	11.4
	20.00	21.03	2.68	12.8
B	0.04	0.034	0.002	4.6
	5.00	5.22	0.84	16.2
	40.00	37.49	3.53	9.4
Li	0.04	0.037	0.006	17.1
	5.00	4.63	0.77	16.6
	40.00	35.39	1.09	3.1
Mn	0.04	0.035	0.003	7.8
	5.00	4.28	0.42	9.7
	40.00	41.02	2.61	6.4
Hg	0.04	0.037	0.006	15.8
	5.00	5.30	0.73	13.7
	40.00	37.66	3.70	9.8
Ni	0.04	0.038	0.007	18.5
	5.00	4.75	0.89	18.7
	40.00	41.22	1.19	2.9
Pb	0.04	0.037	0.006	15.9
	5.00	4.57	0.66	14.4
	40.00	36.58	2.02	5.5
Cu	0.04	0.037	0.006	16.7
	5.00	5.17	0.59	11.4
	40.00	44.75	1.60	3.6
Se	0.04	0.041	0.004	9.0
	5.00	5.40	0.56	10.4
	40.00	42.71	0.75	1.8
Sn	0.04	0.034	0.001	3.9
	5.00	4.31	0.26	6.1
	40.00	35.53	2.07	5.8
Zn	0.04	0.034	0.003	9.1
	5.00	5.58	0.33	5.9
	40.00	43.79	2.31	5.3
Ca	0.20	0.189	0.026	13.6
	10.00	11.39	0.70	6.2
	100.00	109.93	6.82	6.2
Mg	0.20	0.216	0.030	13.7
	10.00	9.13	0.61	6.7
	100.00	107.14	1.37	1.3
K	0.20	0.192	0.022	11.3
	10.00	10.31	1.35	13.1
	100.00	98.81	2.17	2.2
Na	0.20	0.188	0.021	11.2
	10.00	9.81	0.93	9.5
	100.00	101.18	5.01	5.0
La	0.02	0.018	0.003	15.1
	1.00	1.03	0.05	4.5
	20.00	22.75	1.35	5.9
Eu	0.02	0.019	0.003	15.5
	1.00	1.05	0.09	8.1
	20.00	22.90	1.10	4.8
Ga	0.02	0.023	0.001	4.9
	1.00	1.10	0.05	4.6
	20.00	22.75	1.55	6.8
Tb	0.02	0.023	0.001	5.8
	1.00	1.05	0.17	15.9
	20.00	21.77	2.55	11.7

**Table 4:** Method validation results for repeatability with calculated analyte concentration (as mean values, mg/kg), standard deviations (SD) and relative standard deviations (RSD%) obtained for rPET samples at 3 spiked levels.

Analyte	Spiked Value (mg/kg)	Calculated Analyte Concentration (mg/kg)	SD (mg/kg)	RSD (%)
Al	0.10	0.108	0.012	11.1
	5.00	4.81	0.29	6.1
	40.00	43.08	4.79	11.1
Fe	0.10	0.103	0.016	15.6
	5.00	4.71	0.41	8.6
	40.00	39.59	3.58	9.1
Sb	0.02	0.021	0.002	7.2
	10.00	9.29	1.11	12.0
	100.00	99.49	5.58	5.6
As	0.02	0.020	0.003	13.1
	1.00	0.97	0.12	12.5
	20.00	19.59	1.49	7.6
Ba	0.02	0.019	0.002	8.9
	1.00	0.97	0.06	6.3
	20.00	19.27	1.80	9.3
Cd	0.02	0.021	0.002	10.8
	1.00	1.04	0.06	5.8
	20.00	17.97	0.79	4.4
Co	0.02	0.022	0.003	11.9
	1.00	1.01	0.07	7.3
	20.00	19.70	1.70	8.6
Cr	0.02	0.022	0.001	4.4
	1.00	1.02	0.06	5.7
	20.00	20.35	1.79	8.8
Ge	0.02	0.022	0.001	6.1
	1.00	1.03	0.07	6.5
	20.00	21.31	3.34	15.7
Mo	0.02	0.023	0.001	4.4
	1.00	1.04	0.05	4.3
	20.00	20.28	1.26	6.2
Sr	0.02	0.021	0.002	7.8
	1.00	1.02	0.11	10.4
	20.00	22.26	2.54	11.4
V	0.02	0.022	0.001	5.0
	1.00	1.05	0.10	9.1
	20.00	21.87	1.74	7.9
B	0.04	0.042	0.005	12.8
	5.00	5.32	0.65	12.2
	40.00	41.47	4.14	10.0
Li	0.04	0.042	0.002	5.4
	5.00	4.92	0.60	12.3
	40.00	43.21	2.59	6.0
Mn	0.04	0.041	0.004	10.0
	5.00	5.11	0.57	11.2
	40.00	42.97	2.35	5.5
Hg	0.04	0.039	0.005	12.2
	5.00	5.18	0.59	11.4
	40.00	42.52	3.95	9.3
Ni	0.04	0.043	0.002	5.5
	5.00	5.47	0.32	5.9
	40.00	43.17	2.72	6.3
Pb	0.04	0.044	0.004	8.1
	5.00	5.06	0.58	11.5
	40.00	39.38	6.06	15.4
Cu	0.04	0.044	0.003	6.9
	5.00	5.12	0.52	10.1
	40.00	42.03	5.10	12.1
Se	0.04	0.045	0.003	5.6
	5.00	4.99	0.48	9.6
	40.00	44.59	2.71	6.1
Sn	0.04	0.044	0.003	7.5
	5.00	4.96	0.41	8.3
	40.00	39.19	2.14	5.5
Zn	0.04	0.043	0.005	11.9
	5.00	5.15	0.50	9.8
	40.00	41.67	4.56	10.9
Ca	0.20	0.217	0.015	7.0
	10.00	10.44	0.58	5.5
	100.00	96.37	4.95	5.1
Mg	0.20	0.217	0.015	6.9
	10.00	9.75	0.90	9.3
	100.00	99.52	4.61	4.6
K	0.20	0.214	0.013	6.1
	10.00	9.96	0.98	9.9
	100.00	100.72	5.19	5.2
Na	0.20	0.211	0.022	10.5
	10.00	9.83	0.91	9.3
	100.00	102.22	9.61	9.4
La	0.02	0.020	0.003	13.2
	1.00	1.01	0.06	6.0
	20.00	19.52	2.68	13.7
Eu	0.02	0.022	0.002	9.1
	1.00	1.07	0.14	12.7
	20.00	19.91	2.37	11.9
Ga	0.02	0.022	0.002	6.9
	1.00	1.04	0.10	10.0
	20.00	20.91	1.38	6.6
Tb	0.02	0.022	0.002	7.7
	1.00	1.01	0.09	8.7
	20.00	20.41	1.81	8.9

## Data Availability

The original contributions presented in the study are included in the article/Supplementary Materials, further inquiries can be directed to the corresponding author.

## Supplementary Materials

The following supporting information can be downloaded at: https://www.mdpi.com/article/10.3390/foods13172716/s1, Figure S1: Spectrum recorded at the instrumental concentration of 1 µg/L, obtained during the calibration of Al, Fe, Sb, As, Ba, Cd, Co, Cr, Ge, Mo, Sr, and V; Figure S2: Spectrum recorded at the instrumental concentration of 50 µg/L, obtained during the calibration of B, Li, Mn, Hg, Ni, Pb, Cu, Se, Sn, and Zn; Figure S3: Spectrum recorded at the instrumental concentration of 100 µg/L, obtained during the calibration of La, Eu, Gd and Tb; Figure S4: Spectrum recorded at the instrumental concentration of 250 µg/L, obtained during the calibration of Ca, Mg, K, Na; Figure S5: Graphical report with IS Recovery; Table S1: List of analytes under examination, classified as heavy metals (HMs) and rare earth elements (REEs), with characteristics related to FCC typology, distinctions between IAS (Intentionally Added Substances) and NIAS (Non-Intentionally Added Substances), Food Contact Material Number (FCM No) and the relevant authorization or compliance with current regulations. Table S2: Values of sensitivity and stability required for successful tuning.
