# Supplementary material for: Advancing Analytical Techniques in PET and rPET: Development of an ICP–MS Method for the Analysis of Trace Metals and Rare Earth Elements
Source: Foods. 2024 Aug 27;13(17):2716. doi: 10.3390/foods13172716 (PMC11395568; doi:10.3390/foods13172716)

Supplementary Materials

Advancing Analytical Techniques in PET and rPET: development of an ICP–MS method for the analysis of Trace Metals and Rare Earth Elements

Fabiana Di Duca [1], Paolo Montuori [1,*], Elvira De Rosa [1], Bruna De Simone [1], Stefano Scippa [1], Giuseppe Dadà [2] and Maria Triassi [1]

[1] Department of Public Health, University "Federico II", Via Sergio Pansini n. 5, 80131 Naples, Italy;
[2] CORIPET Consorzio Volontario, Via S. Maurilio n. 23, 20123 Milan, Italy
* Correspondence: pmontuor@unina.it

Table S1. List of analytes under examination, classified as heavy metals (HMs) and rare earth elements (REEs), with characteristics related to FCC typology, distinctions between IAS (Intentionally Added Substances) and NIAS (Non-Intentionally Added Substances), Food Contact Material Number (FCM No) and the relevant authorization or compliance with current regulations.

Analyte		FCC type	FCM No [3]	Authorised [4]
HMs	Al	*Co-catalyst or residual contaminant* (NIAS)	-	Authorised as additive
	Sb	*Catalyst* (IAS)	398	Authorised as additive or polymer production aid
	As	*Residual contaminant* (NIAS)	-	Not Authorised*
	Ba	*Residual contaminant* (NIAS)	-	Authorised
	B	*Residual contaminant* (NIAS)	-	-
	Cd	*Residual contaminant* (NIAS)	-	Not Authorised*
	Ca	*Residual contaminant* (NIAS)	-	-
	Co	*Residual contaminant* (NIAS)	-	Authorised
	Cr	*Residual contaminant* (NIAS)	-	Not Authorised*
	Fe	*Residual contaminant* (NIAS)	-	Authorised
	Ge	*Co-catalyst or residual contaminant* (NIAS)	-	-
	Li	*Residual contaminant* (NIAS)	-	Authorised
	Mg	*Residual contaminant* (NIAS)	-	Authorised
	Mn	*Residual contaminant* (NIAS)	-	Authorised
	Hg	*Residual contaminant* (NIAS)	-	Not Authorised*
	Mo	*Residual contaminant* (NIAS)	-	Authorised
	Ni	*Residual contaminant* (NIAS)	-	Not Authorised*
	Pb	*Residual contaminant* (NIAS)	-	Not Authorised*
	K	*Residual contaminant* (NIAS)	-	Authorised
	Cu	*Residual contaminant* (NIAS)	-	Authorised
	Se	*Residual contaminant* (NIAS)	-	-
	Na	*Residual contaminant* (NIAS)	-	Authorised
	Sn	*Residual contaminant* (NIAS)	-	Authorised

	Sr	*Residual contaminant* (NIAS)	-	-
	V	*Residual contaminant* (NIAS)	-	-
	Zn	*Residual contaminant* (NIAS)	-	Authorised
REEs	La	*Residual contaminant* (NIAS)	-	Authorised
	Eu	*Residual contaminant* (NIAS)	-	Authorised
	Gd	*Residual contaminant* (NIAS)	-	Authorised
	Tb	*Residual contaminant* (NIAS)	-	Authorised

*These metals are not included in Annex I of the Commission Regulation (EU) 2020/1245 and they are not authorised to be used in FCMs.

Table S2. Values of sensitivity and stability required for successful tuning.

**Parameter**	**Signal**	**Required value**
Sensitivity	Intensity ^{115}In	≥220.000 cps
Sensitivity	Intensity ^{7}Li	≥55.000 cps
Sensitivity	Intensity ^{59}Co	≥90.000 cps
Sensitivity	Intensity ^{238}U	≥300.000 cps
Sensitivity	Intensity ^{209}Bi	≥150.000 cps
Sensitivity	Intensity ^{140}Ce.^{16}O/^{140}Ce	≤ 0.03
Sensitivity	Intensity ^{137}Ba^{2+}/^{137}Ba	≤ 0.05
Stability	^{7}Li	≤ 2
Stability	^{59}Co	≤ 2
Stability	^{238}U	≤ 2
Stability	^{209}Bi	≤ 2
Stability	^{115}In	≤ 2

Figure S1. Spectrum recorded at the instrumental concentration of 1 μg/L, obtained during the calibration of Al, Fe, Sb, As, Ba, Cd, Co, Cr, Ge, Mo, Sr, and V.

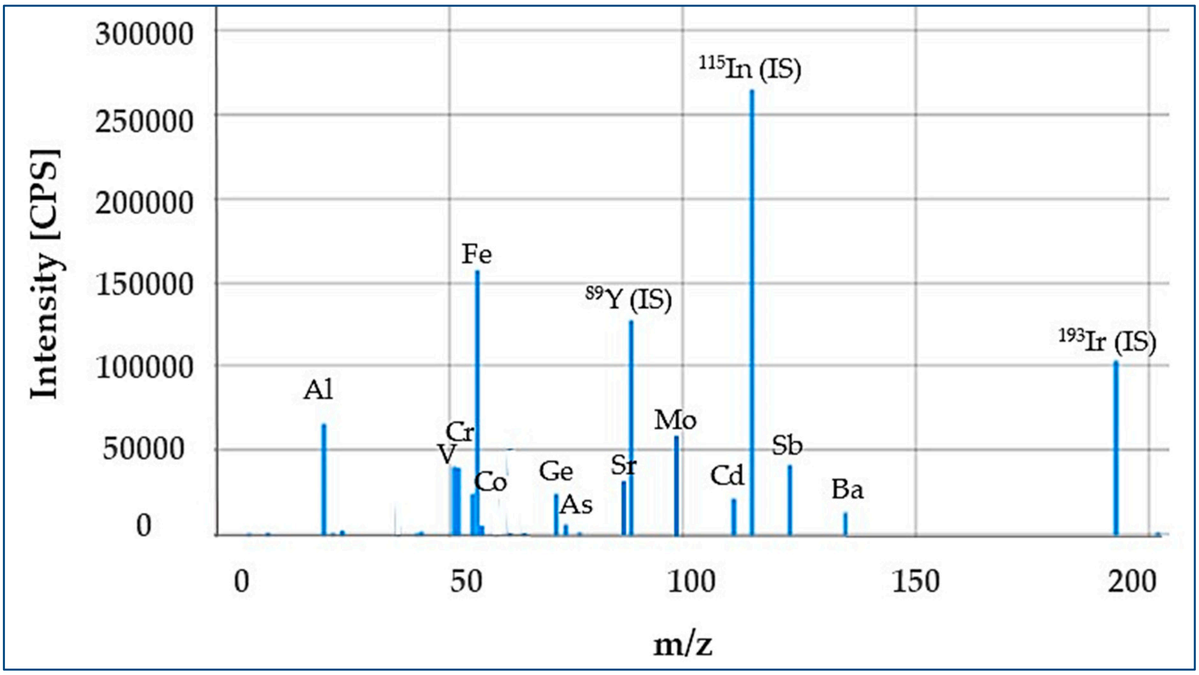

Figure S2. Spectrum recorded at the instrumental concentration of 50 μg/L, obtained during the calibration of B, Li, Mn, Hg, Ni, Pb, Cu, Se, Sn, and Zn.

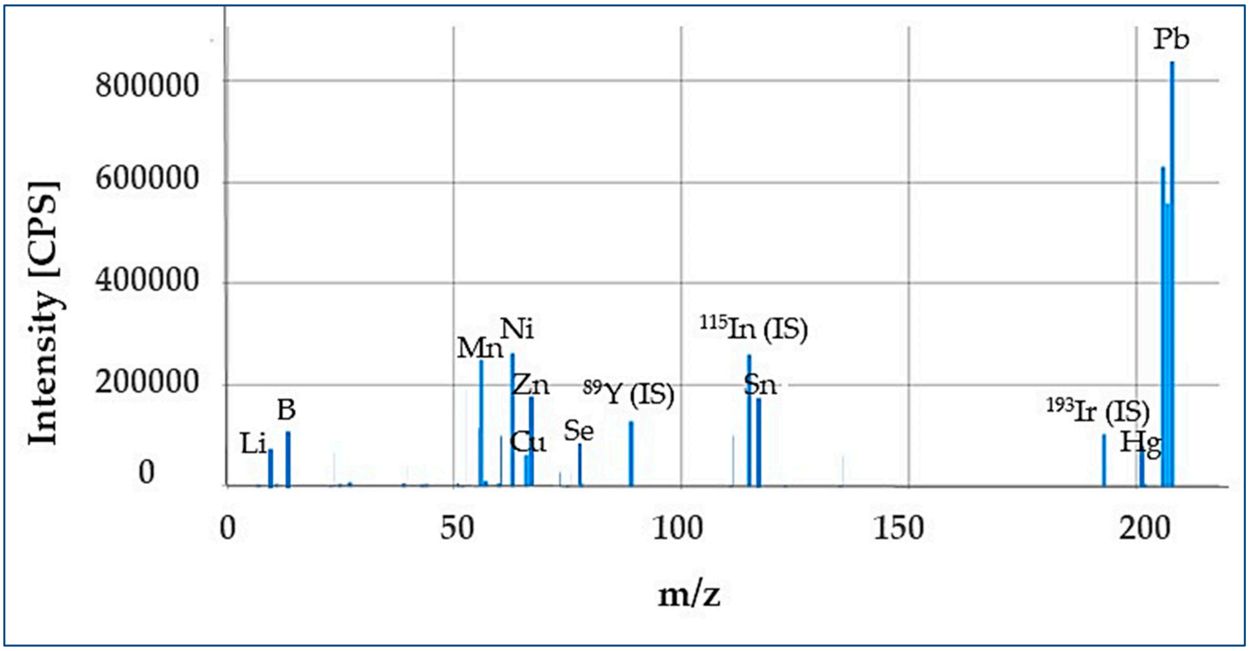

Figure S3. Spectrum recorded at the instrumental concentration of 100 µg/L, obtained during the calibration of La, Eu, Gd and Tb.

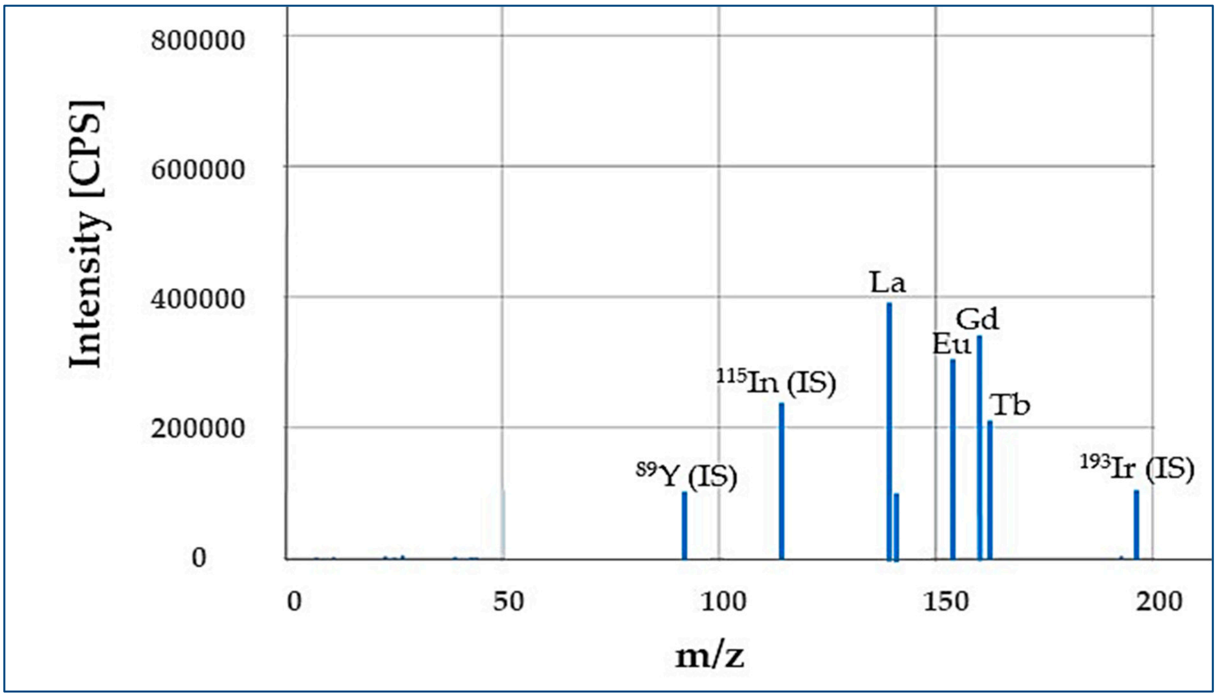

Figure S4. Spectrum recorded at the instrumental concentration of 250 µg/L, obtained during the calibration of Ca, Mg, K, Na.

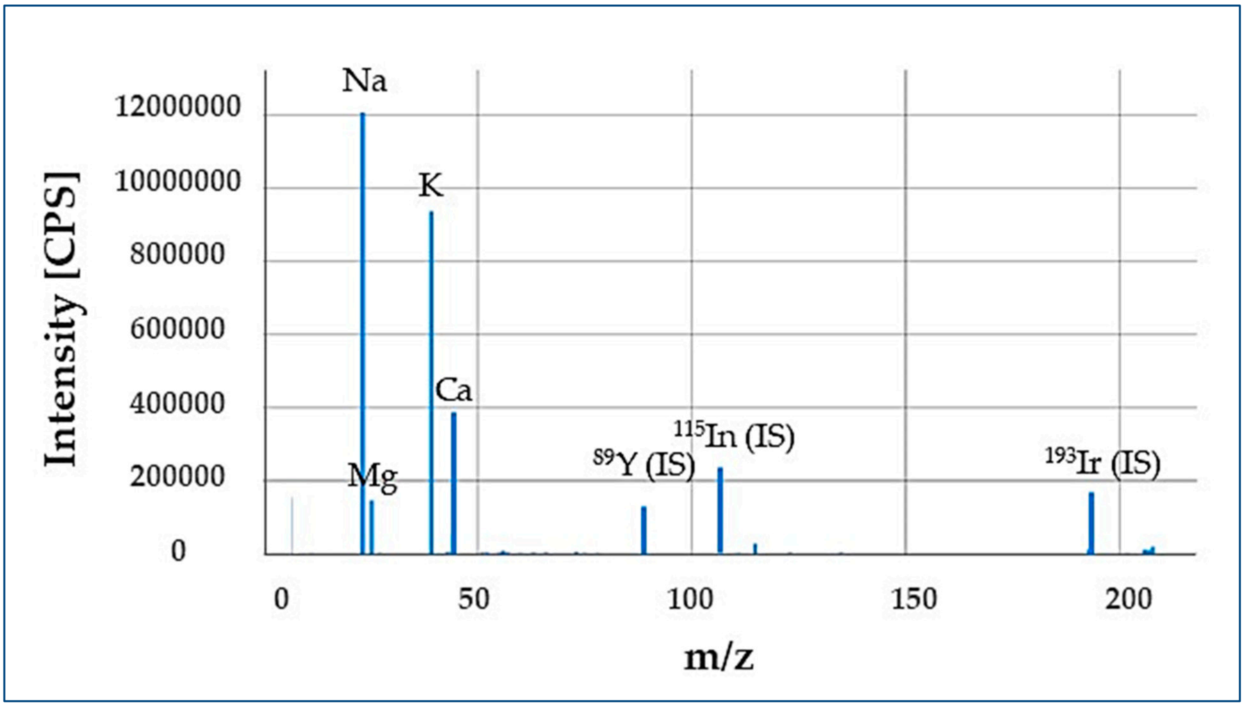

Figure S5. Graphical report with IS Recovery.

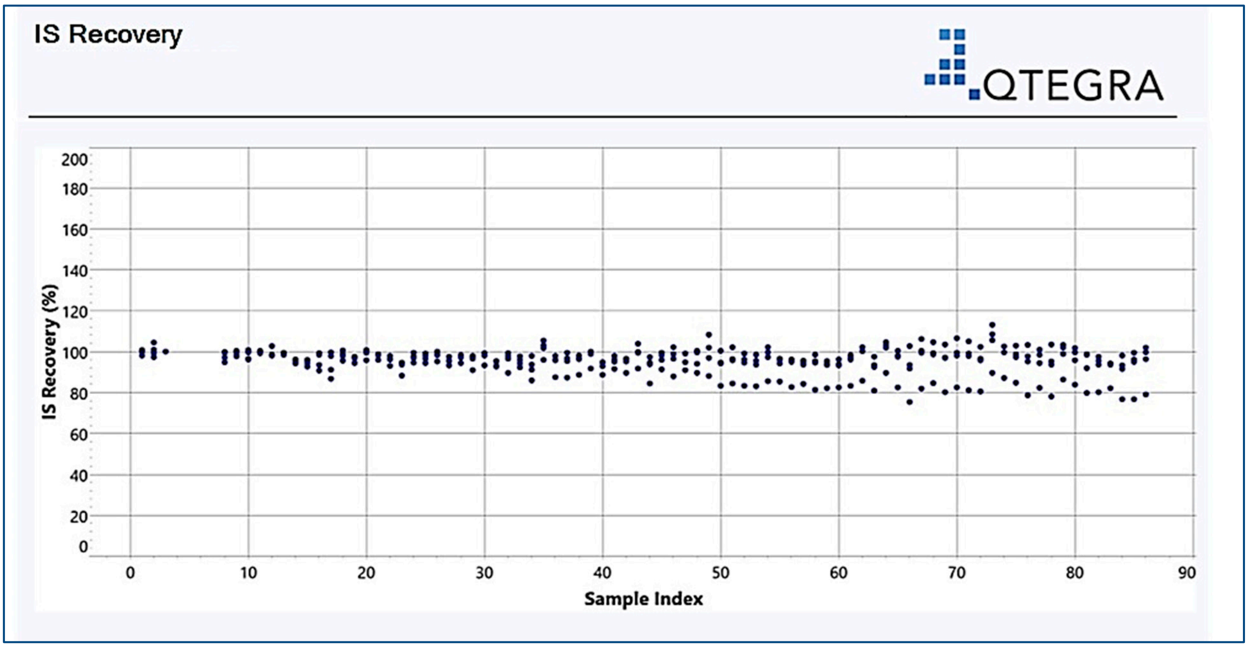

Supplement: Supplementary file 1 [file foods-13-02716-s001.zip › foods-3148199-supplementary.pdf]
